# Cutaneous Manifestations in Patients with Beta-Thalassemia Major

**DOI:** 10.34763/jmotherandchild.20232701.d-23-00047

**Published:** 2023-10-08

**Authors:** Zunaira Zulfiqar, Ayesha Kanwal, Manahil Chaudhry, Muhammad Aadil, Sehrish Qaiser, Ayesha Malik, Mohammad Abdullah

**Affiliations:** Department of Paediatrics, CMH Lahore Medical College & Institute of Dentistry, Lahore, Pakistan; Department of Dermatology, CMH Lahore Medical College & Institute of Dentistry, Lahore, Pakistan; Department of Emergency Medicine, Hameed Latif Hospital, Lahore, Pakistan; Department of Acute Medicine, Russells Hall Hospital, Dudley, United Kingdom; Department of Medicine, CMH Lahore Medical College & Institute of Dentistry, Lahore, Pakistan; Department of Accidents & Emergency, Barking Havering Redbridge University Hospitals, London, United Kingdom; Department of Stroke Medicine, Russells Hall Hospital, Dudley, United Kingdom

**Keywords:** Beta-thalassemia, hyperferritinemia, pruritus, xerosis, cutaneous manifestations

## Abstract

**Background:**

Beta-thalassemia major is a transfusion-dependent thalassemia. Both ongoing disease-related inflammatory processes and chronic transfusions lead to iron overload, which is depicted by hyperferritinemia. We aimed to report the prevalence of various dermatological manifestations in beta-thalassemia major patients and their relationship with serum ferritin levels.

**Material and methods:**

This was a cross-sectional study conducted over a period of six months. Beta-thalassemia major patients were consecutively enrolled and examined by a dermatologist who charted any skin conditions, if present. A blood sample was also taken at the same time to check for the serum ferritin levels. Data was analysed using SPSSv25.

**Results:**

A total of 113 patients were included in the study. The mean age of the cohort was 9.32 ± 4.54 years. The mean ferritin level for the cohort was 3334 ± 1676 micrograms per litre. Cutaneous manifestations were seen in 89.4% (n = 101) patients with the common ones namely xerosis (44.2%), freckles (39.8%) and pruritus (44.2%). We noted that serum ferritin levels were significantly higher in those with freckles (p = 0.00288). The cause of pruritus does not appear to be jaundice (p = 0.973). Lastly, number of skin conditions were higher in those with onset of blood transfusions at age less than one year (p = 0.0011).

**Conclusion:**

Dermatological manifestations are a frequently encountered problem in beta-thalassemia major patients. It is important to examine these patients for various skin disorders periodically as this can help improve their quality of life and reduce dermatological-associated morbidity.

## Introduction

Thalassemias are a group of blood disorders that affect haemoglobin (Hb) polypeptide chains present in red blood cells (RBCs). In beta-thalassemia patients, the beta polypeptide chain is abnormal due to various single gene mutations. This leaves free alpha chains in the RBCs, which clump together and damage the cell membrane resulting in haemolysis. This haemolysis of mature erythrocytes leads to an ‘ineffective erythropoiesis’, eventually resulting in anaemia. Therefore, beta-thalassemia major, and occasionally intermedia, are transfusion dependent.

Globally, it is estimated that around 23,000 children are born with beta-thalassemia major every year, and most of these births occur in Mediterranean, African and Southeast Asian populations [1]. In an estimate of disease burden, it was noted that up to 9,000 children are born with beta-thalassemia major each year in Pakistan [2]. With more advance treatment options, the life expectancy of these children has been prolonged. This allows us to better understand complications that result from the disease process itself, as well as those brought about by the treatment, which includes chronic transfusions and chelation therapy. Chronic transfusions and ongoing inflammatory processes lead to a buildup of iron, also known as haemosiderosis, which is reflected as hyperferritinemia. Haemosiderosis, in turn, can affect several body organs including skin, and this is infrequently studied. Therefore, we aimed to look at the various dermatological manifestations prevalent in beta-thalassemia major patients and their individual relationship with serum ferritin levels.

## Material and methods

This study was carried out over a period of six months in the paediatrics department after the approval from the hospital's ethical review committee. All patients attending the inpatient and outpatient paediatric clinics with a diagnosis of beta-thalassemia major were consecutively invited to participate in the study and informed consent was sought from the parent(s). All patients with skin conditions prior to the diagnosis of beta-thalassemia, all those having skin manifestations with diagnostic uncertainty, and all those who did not consent were excluded.

After logging the age and gender of each patient, the medical history pertinent to beta-thalassemia was sought. This included the age at the time of diagnosis, age of onset of blood transfusions and whether the patient received chelation therapy. If on chelation, the type of medication used was also noted. All patients then went on to be examined by a dermatologist, for various skin conditions which were charted. In addition, each participant's serum ferritin level was also obtained on the day of inclusion.

Descriptive statistics were drawn and are presented as mean ± SD and percentages. Qualitative data was compared using the chi-square test or Fischer Exact test. Quantitative, non-parametric data were compared using the Mann–Whitney test. A p-value ≤ 0.05 was considered as statistically significant.

## Results

A total of 120 patients with beta-thalassemia major were invited to participate; of these a final 113 were included after meeting the criteria and being examined dermatologically. Amongst them were 42 girls (37.2%) and 71 boys (62.8%) between the ages of 2 and 19 years. The mean age of the cohort was 9.32 ± 4.54 years. The mean ferritin level for the cohort was 3334 ± 1676 micrograms per litre. When separated according to the age at the time of diagnosis of beta-thalassemia major, it was found that 69.9% of our patients were diagnosed before the age of one year.

There are three types of chelation therapies that are available for beta-thalassemia patients, namely, deferasirox, desferrioxamine and deferiprone, and 93.8% (n = 106) patients in our cohort were receiving chelation therapy at the time of the study, from which 61.3% (n = 65) patients were on a single agent and 36.7% (n = 41) were receiving chelation therapy with more than one type of drug.

**Figure 1.**
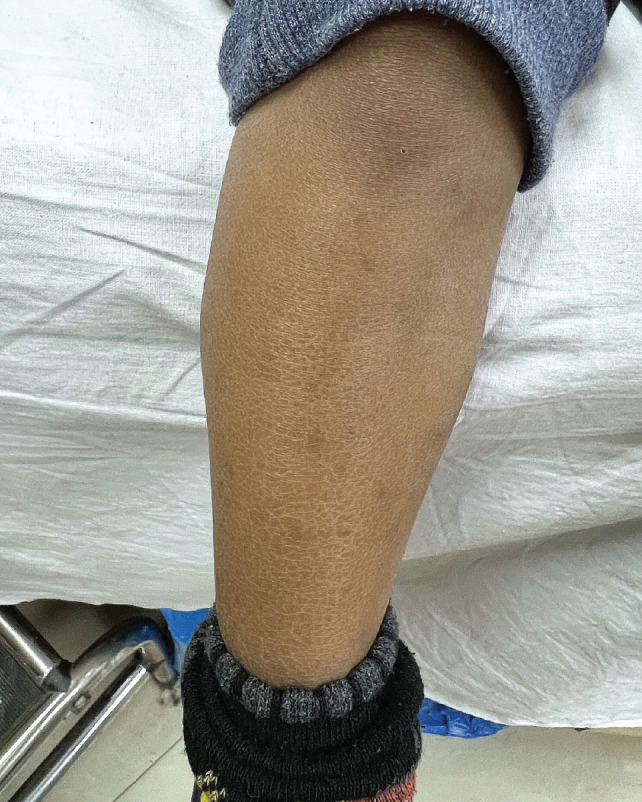
Xerosis – extensor surface of leg shows dry skin.

When examined for dermatological manifestations, 10.6% (n = 12) did not have any skin conditions identified, and 89.4% (n = 101) patients were diagnosed with one or more skin condition as follows: xerosis (44.2%), freckles (39.8%), pruritus (44.2%), pityriasis alba (11.5%), jaundice (7%), injection site marks (18.6%), miliaria (8.8%), tinea versicolor (13.3%), eczema (9.7%) and herpes (1.8%), as elaborated in Fig 1. The most common skin conditions were xerosis and pruritus.

**Table 1.** Relationship between different skin disorders and mean serum ferritin levels.

**Skin disorder (n)**			**Mean ferritin levels ± standard deviation**	**P-value**
Pruritis	Present	50	3419 ± 1733	0.682
	Absent	63	3266 ± 1641	
Xerosis	Present	50	3615 ± 1695	0.0615
	Absent	63	3111 ± 1641	
Eczema	Present	11	3848 ± 1680	0.211
	Absent	102	3278 ± 1675	
Pityriasis alba	Present	13	2658 ± 1277	0.142
	Absent	100	3421 ± 1707	
Injection site marks	Present	21	3155 ± 1312	0.857
	Absent	92	3374 ± 1752	
Herpes	Present	2	4180 ± 863	0.223
	Absent	111	3356 ± 1681	
Tinea versicolor	Present	15	2801 ± 1662	0.134
	Absent	98	3415 ± 1672	
Miliaria	Present	10	3682 ± 1694	0.373
	Absent	103	3300 ± 1679	
Jaundice	Present	8	3449 ± 1934	0.818
	Absent	105	3325 ± 1665	
Freckles	Present	45	4013 ± 1976	**0.00288**
	Absent	68	2884 ± 1272	

Statistically significant values are in bold.

**Figure 2.**
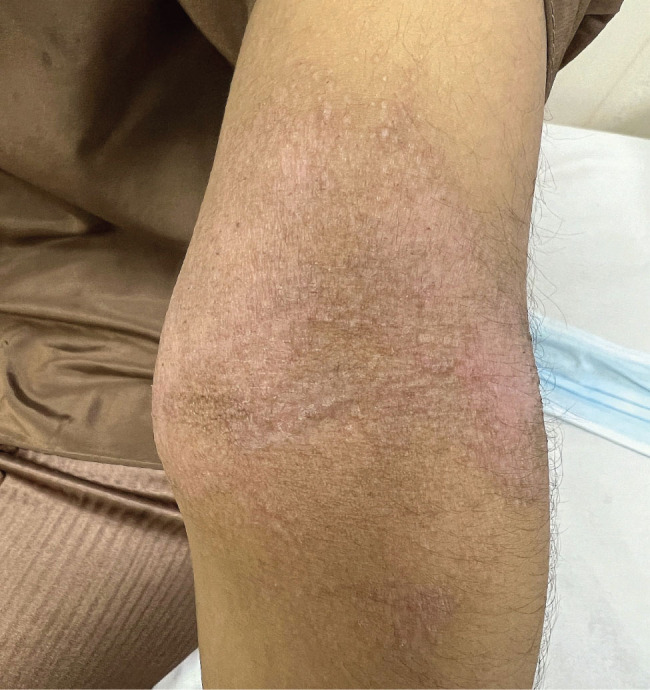
Eczema – well-defined erythematous scaly plaque with wrinkling of skin involving cubital fossa of arm, along with some excoriation marks.

**Figure 3.**
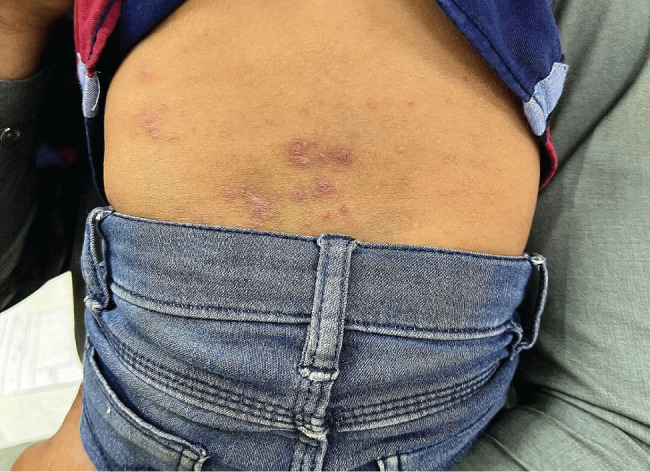
Eczema – multiple ill-defined erythematous and excoriated papule and plaques over dry wrinkled hyperpigmented skin of lower back.

Table 1 shows the correlation between mean serum ferritin levels and various skin conditions. The serum ferritin levels were significantly higher in those with freckles (p = 0.00288), while all other identified skin conditions were insignificantly associated with ferritin levels. The normal range of ferritin is 25 to 200 nanogram per millilitres (ng/mL) for newborns, 200 to 600 ng/mL at 1 month old, 50 to 200 ng/mL at 2 to 5 months old, 7 to 142 ng/mL for children 6 months to 15 years, 12 to 300 ng/mL for adult males and 10 to 150 ng/mL for adult females [3, 4].

We checked for a correlation between the age of onset of blood transfusion (BT), that is, before or after one year of age, and the number of skin lesions, and we noted that patients who started receiving BT before the age of one, had more incidence of having one or more skin conditions (p = 0.0011). For this analysis, we used the Fisher Exact test, and patients in whom BT had not been started at the time of the study were excluded.

Lastly, we studied for any relation between pruritus and the presence of jaundice and found this relation to be statistically insignificant (p = 0.973).

## Discussion

Beta-thalassemia patients undergo multiple transfusions during their lifetime, and one unit of red blood cells transfuses about 250 mg of iron, whereas the maximum excreted amount by the body is 1 mg of iron per day [5]. In addition, there is ongoing ineffective erythropoiesis, which means reduced consumption of iron. Simultaneously, there is also increased absorption of iron from the gut, and the net effect is an overall increase in the iron load in the body or haemosiderosis [6]. This iron overload is depicted by hyperferritinemia in the blood, as ferritin is the primary storage form of iron.

Chronically transfused patients have a higher propensity for haemosiderosis. Therefore, we tested the relationship between age of commencement of transfusions and number of skin disorders and found that those patients who were started on blood transfusions before the age of one year (more repeated transfusions) had a more incidence of having a skin condition (p = 0.0011) than those who were started on transfusions after the age of one year.

We further tested the correlation between serum ferritin levels with each skin condition. We found that the presence of freckles was significantly associated with higher serum ferritin levels (p = 0.00288), while other skin conditions, namely, xerosis, pruritus, pityriasis alba, jaundice, injection site marks, miliaria, tinea versicolor, eczema and herpes, had an insignificant relationship with the serum ferritin levels. Fahmey et al. also reported a statistically significant relationship between serum ferritin levels and freckles (p < 0.05), along with scars, hyperpigmentation and xerosis [7]. These set of results propose that the risk of developing iron overload-associated dermatoses is high in patients with beta-thalassemia major.

Overall, the most prevalent skin findings in our study were pruritus (44.2%) and xerosis (44.2%), proceeded by freckles (39.8%). Similarly, a study by El-Dash et al. also reported the highest prevalence of xerosis and pruritus in their cohort of beta-thalassemia major patients [8]. Dogramaci et al. also reported matching findings with our study, that is, pruritus and xerosis, being the commonest [1]. It is theorised that iron deposition in the cutaneous tissues stimulates the release of histamine from tissue mast cells [9], which can explain the common occurrence of pruritus in these patients.

One other commonly known trigger of pruritus is jaundice (hyperbilirubinemia), which was seen in 7% of our cohort; however, the two were insignificantly related. This means that the pruritus seen in these patients is likely not related to the presence of hyperbilirubinemia. Xerosis, which is fissuring of the skin due to excessive water loss from the epidermis remains yet unexplained in thalassemia patients. Thirteen patients in our study were found to have a skin condition called pityriasis alba, which have also been reported previously in beta-thalassemia major patients [1, 7]. These hypopigmented patches are a few centimetres in diameter, more pronounced in people of colour, and are seen on the upper body and face. Hypopigmented areas lack melanin, whose production requires tyrosinase, which is a melanogenic-copper enzyme [10]. Beta-thalassemia is reported to be associated with reduced copper levels, and this can theorise why pityriasis alba can occur more frequently in these patients [11].

Another skin condition is tinea versicolor, which was seen in 15 (13.3%) patients in our study, and can occur because of impaired immune responses in poly-transfused thalassaemia patients [12], resulting in overgrowth of the causative fungus, Malassezia.

A limitation of the study is the localisation of the sample to a single study centre. This limits us in terms of the number of examined patients. We also wished to have improved the examination of the skin manifestations by having the expert opinions of more than one dermatologist to ensure no finding was missed. Lastly, it may be difficult to differentiate skin manifestations that may have other underlying causes unrelated to beta-thalassemia major and its complications.

## Conclusion

1. Dermatological manifestations are frequently observed in patients of beta-thalassemia major; the most seen ones are pruritus, xerosis and freckles.
2. We noted freckles to be associated with higher serum ferritin levels, and this calls for further studies to assess ferritin levels as a biological prognostic marker for dermatological-associated morbidity in thalassemia patients.
3. It is important to examine these patients for various skin disorders periodically as this can help improve their quality of life. One way is to involve a dermatologist in the multi-disciplinary managing team.
